# Mechanistic Insight Into the Interaction Between *Helicobacter pylori* Urease Subunit α and Its Molecular Chaperone Hsp60

**DOI:** 10.3389/fmicb.2019.00153

**Published:** 2019-02-05

**Authors:** Huilin Zhao, Yulong Wu, Zheng Xu, Ran Ma, Yunfei Ding, Xuelian Bai, Qianyu Rong, Ying Zhang, Boqing Li, Xiaofei Ji

**Affiliations:** ^1^Department of Pathogenic Biology, School of Basic Medical Sciences, Binzhou Medical University, Yantai, China; ^2^School of Pharmacy, Binzhou Medical University, Yantai, China; ^3^Clinical Medicine Laboratory, Binzhou Medical University Hospital, Binzhou, China

**Keywords:** *Helicobacter pylori*, urease, Hsp60, chaperone, protein–protein interaction

## Abstract

*Helicobacter pylori* is the etiologic agent in a variety of gastroduodenal diseases. As its key pathogenic factors, both urease and Hsp60 play important roles in the pathogenesis of *H. pylori*. Previous studies have suggested that there is close relationship between urease and Hsp60, which implied that Hsp60 may act as a chaperone in urease stabilization and assembly. However, how these two proteins interact remains unclear. In this study, the impact of Hsp60 on urease activity of *H. pylori* lysate was first detected to confirm the interaction between urease and Hsp60. Pull-down assays further indicated that Hsp60 could bind to UreA subunit but not UreB. Then, the 3D structure of Hsp60 was modeled using I-TASSER to simulate the binding complex with UreA by molecular docking. The results showed that UreA is a perfect fit for the cavity of Hsp60. Analysis of the resulting model demonstrated that at least seven residues of UreA, located on two interfaces, participate in the interaction. Site-directed mutagenesis of these potential residues showed reduced affinity with Hsp60 than the wild type UreA through surface plasmon resonance (SPR) experiments, and D68 appears to have an important role in the affinity. Further analysis also showed that mutation of E25 and K26 caused a more rapid association and dissociation than with wild UreA, implying that they have roles in stabilizing the interaction complex. These affinity comparisons suggested that the interfaces predicted by molecular docking are credible. Our study indicated a direct interaction between Hsp60 and urease and revealed the binding interfaces and key residues involved in the interaction. These results provide further evidence for the chaperone activity of Hsp60 toward urease and lay a foundation to better understand the maturation mechanism of urease in *H. pylori*.

## Introduction

*Helicobacter pylori* is a Gram negative bacterium that colonizes the gastric mucosa (Marshall and Warren, 1984) of half the adult population worldwide (Eusebi et al., 2014). It is usually related to peptic ulcers and is a major risk factor for the development of gastric cancer (Uemura et al., 2001). Urease is one of the most important pathogenic factors for *H. pylori*, which accounts for 10–15% of total protein of the bacterium by weight (Ha et al., 2001). Up to 30% of urease is located on the surface of *H. pylori* (Dunn et al., 1997). *In vitro* studies have indicated that successful colonization by *H. pylori* in the acidic stomach environment requires active external urease, which catalyzes the hydrolysis of urea to ammonia and carbon dioxide, generating a hospitable locale for the bacterium. *H. pylori* can then safely pass through the gastric fluid and mucus layer to reach the neutral mucosal surface (Khan et al., 2009). Therefore, the activity and stability of urease is essential for colonization by *H. pylori* in the human stomach.

*Helicobacter pylori* urease is composed of two structural proteins, α and β subunits, where the β subunit is 60 kDa and the α subunit is approximately 30 kDa. In 2001, the structure of *H. pylori* urease was resolved by Ha et al. via x-ray crystallography. They found that the cluster of 12 active dimers [4(αβ)_3_] in the supramolecular assembly is critical for the activity of the enzyme in an acid environment (Ha et al., 2001). However, how the external urease maintains its stability before the assembly of the 12 subunits remains unclear. It has been speculated that a chaperone participates in this process.

Hsp60 is a molecular chaperone that exists widely in both prokaryotic and eukaryotic organisms and plays important roles in protein homeostasis by mediating protein folding and assembly (Okamoto et al., 2017). It is highly conserved and shows high similarity in amino acid sequences between bacteria and other higher organisms (Dunn et al., 1992; Suerbaum et al., 1994). The structure of Hsp60 in *Escherichia coli* (named GroEL) was resolved in 1994, which showed that seven monomers are arranged in a ring. Two rings are arranged back-to-back, forming a 14 subunit porous cylinder that acts as a chaperone (Braig et al., 1994). A large amount of evidence demonstrates that a part of Hsp60 may be expressed on the bacterial cell surface and is closely related to pathogenesis in some bacterial species (Bajzert and Stefaniak, 2015).

*Helicobacter pylori* produces a large amount of Hsp60. As a virulence factor, its role in the adhesion of *H. pylori* to host cells has been extensively reported (Yamaguchi et al., 1997; Kamiya et al., 1998). Moreover, Hsp60 has also been reported to participate in immune protection as an extracellular antigen of *H. pylori* (Yamaguchi et al., 2000; Bai et al., 2003). Although it has different oligomeric forms to *E. coli* GroEL (*H. pylori* Hsp60 usually exists as dimers and tetramers while *E.coli* Hsp60 preferentially forms heptamers) (Lin et al., 2009), *H. pylori* Hsp60 is also expected to act as a molecular chaperone (Austin et al., 1992; Suerbaum et al., 1994). This was confirmed by Mendoza et al. (2017) where they showed that *H. pylori* Hsp60 has chaperone activity that suppresses the acid-induced aggregation of alcohol dehydrogenase (ADH) under moderately acidic conditions *in vitro*. However, it is not clear how many or what proteins are the substrate proteins of Hsp60 in *H. pylori*.

The close relationship between urease and Hsp60 in *H. pylori* was first recognized after Hsp60 was frequently co-purified with *H. pylori* urease (Dunn et al., 1991; Evans et al., 1992). It was then found that the co-expression of Hsp60 with urease in *E. coli* greatly increased the activity of urease (Suerbaum et al., 1994). Moreover, the supramolecular assembly of Hsp60 is very similar to native urease polymers (Austin et al., 1992; Ha et al., 2001). All these points of evidence suggest that Hsp60 acts as a molecular chaperone for urease.

In this manuscript, we detected the interaction between *H. pylori* Hsp60 and urease using both urease activity and pull-down assays. An interaction model was simulated by molecular docking to analyze the interface of the complex and the amino acids playing key roles in this interaction. Site directed mutants of these potential key residues on urease were constructed and finally the affinities of these mutants to Hsp60 were compared to the wild type urease using SPR to verify roles of these amino acids in the interaction. Our results show the interaction between Hsp60 and urease at both biochemical and molecular levels, which lay a foundation for further understanding of the assembly mechanism of urease in *H. pylori*.

## Materials and Methods

### Bacterial Strains and Growth Conditions

*Helicobacter pylori* 26695 (ATCC700392) was used as a template for amplification of the urease α subunit (UreA), β subunit (UreB), and Hsp60 genes. *E. coli* DH5α and BL21(DE3) were used as a cloning strain and expression strain, respectively, for heterologous expression of the above-mentioned genes. pEASY-Blunt-E1 (Transgen) and pET-22b(+) (Novagen) were used as plasmids for the construction of expression vectors. *H. pylori* was cultured using CM0935 Campylobacter agar base (OXOID) supplemented with sheep’s blood (10%) at 37°C under microaerobic conditions containing 85% N_2_, 10% CO_2_, and 5% O_2_. *E. coli* strains were routinely cultured at 37°C in Luria–Bertani medium (OXOID). Ampicillin was used at the concentrations of 100 mg/l when needed for the selection of *E. coli* transformants.

### Expression and Purification of Hsp60, UreA-His, and UreB-His

Genomic DNA of *H. pylori* 26,695 was extracted with a bacterial DNA extract kit (Tiangen), according to the manufacturer’s instructions. A DNA fragment encoding Hsp60 (from M1 to M546) was amplified by PCR with primers H-F and H-R (Table 1). The obtained fragment was ligated into the vector pEASY-Blunt-E1 to construct the Hsp60 expression vector. The recombinant plasmid was then transformed into *E. coli* BL21(DE3), which was then grown on media containing ampicillin overnight. Transformants were picked into LB-ampicillin broth and grown at 37°C to an optical density at 600 nm (OD600) of 1.0. Expression of Hsp60 was induced with 0.25 mM isopropyl β-D-thiogalactopyranoside (IPTG) at 100 rpm, 20°C overnight. Cells were harvested by centrifugation at 11,000 × *g*, 4°C for 5 min, washed by Tris-HCl (50 mM, pH8.0) twice and resuspended in the same buffer. Crude recombinant Hsp60 with a 6 × His tag on its C terminus was obtained by disruption of the cells using sonication and then purified using a Ni-NTA his bind resin (Transgen). Hsp60 with a higher purity was obtained by gel filtration chromatography using Sephadex G-75 resin (GE Healthcare). The purity of the eluate was analyzed by SDS-PAGE.

**Table 1:** Primers used in this study.

Primer	Description or sequence^a^
H-F	ATGGCAAAAGAAATCAAATTTTC
H-R	TTACATCATGCCACCCATGCC
UA-GN-F	GTCTCGGAATTCAAACTCACCCCAAAAGAGTTAG
UA-GN-R	GCTGCGCTCGAGTTACTCCTTAATTGTTTTTACATAG
UB-GN-F	GCACGGGAATTCAAAAAGATTAGCAGAAAAGAAT
UB-GN-R	CATGGCCTCGAGCTAGAAAATGCTAAAGAGTTGC
UA-GC-R	CGGTCGGAATTCCTCCTTAATTGTTTTTACATAGT
UB-GC-R	TCGTAGGAATTCGAAAATGCTAAAGAGTTGCGCCAA
GST-F	GCTACGGAATTCATGTCCCCTATACTAGGTTA
GST-R	TGATGGCTCGAGTTAATCCGATTTTGGAGGATGGT
UA-H-F	CGAAGTCCCATATGAAACTCACCCCAAAAGAGTTAG
UA-H-R	GCTGCGCTCGAGCTCCTTAATTGTTTTTACATAG
UB-H-F	CGAAGTCCCATATGAAAAAGATTAGCAGAAAAGAAT
UB-H-R	CATGGCCTCGAGGAAAATGCTAAAGAGTTGC
UreA-Y15A-F	CGAAGTCCCATATGAAACTCACCCCAAAAGAGTTA GACAAGTTGATGCTCCAC**GC**TGCTGG
UreA-K22A-F	CGAAGTCCCATATGAAACTCACCCCAAAAGAGTTAG ACAAGTTGATGCTCCACTATGCTGGAGAATTGGCT AAA**GCT**CGCAAAG
UreA-R23A-F	CGAAGTCCCATATGAAACTCACCCCAAAAGAGTTAG ACAAGTTGATGCTCCACTATGCTGGAGAATTGGCTA AAAAA**GC**CAAAGAAAAAGGC
UreA-E25A-F	CGAAGTCCCATATGAAACTCACCCCAAAAGAGTTAG ACAAGTTGATGCTCCACTATGCTGGAGAATTGGCTA AAAAACGCAAAG**CT**AAAGGC
UreA-K26A-F	CGAAGTCCCATATGAAACTCACCCCAAAAGAGTTAGA CAAGTTGATGCTCCACTATGCTGGAGAATTGGCTAA AAAACGCAAAGAA**GCT**GGCATTAAGC
UreA-K66A-F	GCGCACTCTTTTA**GCT**CCGGATGATGTGATGG
UreA-K66A-R	CCATCACATCATCCGG**AGC**TAAAAGAGTGCGC
UreA-D68A-F	GCACTCTTTTAAAACCG**GCT**GATGTGATGGATGGC
UreA-D68A-R	GCCATCCATCACATC**AGC**CGGTTTTAAAAGAGTGC

^a^*Restriction sites on the primers are underlined. Mutated bases are marked with bold font and underlined with double lines*.

Genes of UreA (from M1 to E238) and UreB (from M1 to F569) subunits were amplified from the genome DNA of *H. pylori* 26695 with primers UA-H-F, UA-H-R and UB-H-F, UB-H-R (Table 1), respectively. After digested with *Nde*I-*Xho*I, the purified fragments were ligated into corresponding sites of vector pET-22b(+) (Novagen). The constructed plasmids were introduced into *E. coli* BL21(DE3) for expression. The transformants selection, induction and the purification of crude protein were performed just as that of Hsp60. Protein concentrations were determined using a BCA protein assay kit (Thermo), according to the manufacturer’s instructions.

### Urease Activity Assay

The activity of urease was obtained by measuring ammonia production using phenol-hypochlorite method as described by Yang et al. (2018). *H. pylori* cells collected from plates were suspended in 50 mM HEPES buffer (pH 7.5) and lyzed by sonication. Then 90 μl of the lysate or the lysate supernatant (after centrifugation at 12,000 × *g* for 20 min to remove the insoluble fractions) was incubated with Hsp60 (100 μl, 0.2 mg/ml dissolved in 50 mM HEPES, pH 7.5) at 37°C for 30 min to make an adequate interaction. The mixture was added with100 μl of urea solution (62.5 mM in the same buffer) and incubated for another 30 min at 37°C, followed by phenol-hypochlorite reaction for determination of released ammonia. The absorbance at 620 nm was measured using a microplate reader (Tecan Infinite^®^ 200 Pro) finally. Samples with boiled Hsp60 substitute for native Hsp60 was used as a control. The impact of Hsp60 on heterologously expressed urease (UreA-His and UreB-His) was also detected using this method, when the lysate of *H. pylori* was changed to a 1:1 mixture of UreA-His and UreB-His with the concentration of 5 μM, respectively.

### Pull-Down Assays

UreA and UreB were also expressed as glutathione S-transferase (GST) fusion proteins (N terminal fusion or C terminal fusion) in pull-down assays. For recombinant UreA and UreB containing an N-terminal GST tag, fragments were amplified with primers UA-GN-F/UA-GN-R and UB-GN-F/UB-GN-R, digested with *Eco*RI-*Xho*I, and inserted into the corresponding sites of the vector pGEX-4T-1 (GE Healthcare). Recombinant UreA and UreB containing a C-terminal GST tag were constructed in two steps. Sequences of UreA and UreB were amplified with primers UA-H-F/UA-GC-R and UB-H-F/UB-GC-R, which were then cloned into *Nde*I and *Eco*RI-digested pET-22b(+) to generate the recombinant vectors. The GST sequence was obtained by PCR from template vector pGEX-4T-1 using primers GST-F and GST-R before digestion with *Eco*RI and *Xho*I. The fragment was then ligated with the recombinant vectors from the previous step to generate plasmids pET-22b-UreA-GST and pET-22b-UreB-GST. These plasmids were transformed into *E. coli* BL21(DE3). Verified transformants were cultivated at 37°C in liquid LB medium supplied with ampicillin overnight. The culture was diluted into fresh medium at 1:100 and incubated for 2 h at 37°C followed by 12 h-incubation at 20°C in the presence of 0.25 mM IPTG. Cell disruption was performed by sonication in Tris-HCl (50 mM, pH 8.0). Recombinant GST fusion proteins in the supernatant were immobilized on glutathione Sepharose 4B beads (GE Healthcare), which were then incubated with 0.4 ml of purified recombinant Hsp60 (1 mg/ml) for 2 h at 4°C. After the supernatant was removed, the pellets were washed four times with Tris-HCl buffer and resuspended in 50 μl of SDS-PAGE sample buffer prior to boiling. Finally, 20 μl of the supernatant was used for Western blotting and the remainder for SDS-PAGE analysis. Monoclonal mouse antibody for His-Tag (Proteintech) and horseradish peroxidase (HRP)-conjugated goat anti-mouse IgG 158 (Cowin Biotech) were used for Western blotting. The GST tag expressed alone was used as a negative control.

### Homology Modeling of Hsp60 in *H. pylori*

A three-dimensional model of Hsp60 was constructed using the I-TASSER protein modeling server^1^. The crystal structure of GroEL from *E. coli* (PDB: 2YNJ) (Bartesaghi et al., 2012), which shares 61% identity with *H. pylori* Hsp60, was used as a template out of the 10 top templates chosen from the LOMETS threading program. The I-TASSER server builds models through an exhaustive process involving automatic template selection, reassembly of aligned regions, unaligned regions (mainly loops) built by ab initio modeling, simulation decoy clustering, energy evaluation and optimization of the hydrogen-bonding network (Yang et al., 2015). Visualization and analysis of the resulting model were performed with PyMOL (Roy et al., 2010).

### Molecular Docking Simulation

The 3D structure of the urease was downloaded from RCSB Protein Data Bank (PDB: 1E9Z) (Ha et al., 2001), while the structure of Hsp60 was constructed as described above. Protein-protein docking in ClusPro server (Kozakov et al., 2017) was used for molecular docking simulations and predicting the binding affinity for UreA and Hsp60. UreA was defined as ligand, and Hsp60 as target. The ligand was rotated with 70,000 rotations. For each rotation, the ligand was translated in *x*, *y*, and *z* axis relative to the receptor on a grid. One translation with the best score was chosen from each rotation. Of the 70,000 rotations, 1000 rotation/translation combinations that have the lowest score was chosen. Then, a greedy clustering of these 1000 ligand positions with a 9 Å C-alpha RMSD radius was performed to find the ligand positions with the most “neighbors” in 9 Å, i.e., cluster centers. The top 10 cluster centers with most cluster members were then retrieved and inspected visually one by one. The one with the lowest binding energy was finally recognized and the intermolecular contacts between UreA with Hsp60 were further evaluated. The docked structures and interface residues were analyzed using MOE v2014.09 (Chemical Computing Group Inc, 2014). Molecular graphics were generated by PyMOL.

### Site-Directed Mutagenesis Construction of UreA-His

According to our analysis, seven residues on the N terminus of UreA, including Y15, K22, R23, E25, K26, K66, and D68, were considered as potential key amino acids in the interaction. Therefore, site-directed mutants of UreA-His were constructed where each residue mentioned above was substituted with an alanine. Mutations of residues close to the N-terminus of UreA, including Y15, K22, R23, E25, and K26, were introduced directly by the forward primers with mutations listed in Table 1 (UreA-Y15A-F, UreA-K22A-F, UreA-R23A-F, UreA-E25A-F, UreA-K26A-F). UA-H-R was used as the reverse primer. Mutations of K66 and D68 were constructed by overlapping extension-PCR with primers UreA-K66A-F/UreA-K66A-R and UreA-D68A-F/UreA-D68A-R, respectively, as well as the terminal primers UA-H-F and UA-H-R. The mutated genes were digested and inserted into *Nde*I and *Xho*I-digested pET-22b(+) and transformed into *E. coli* BL21(DE3). After confirmation by restriction digestion and nucleotide sequencing, all mutants were expressed and purified as for wild-type UreA-His.

### Circular Dichroism (CD) Spectra Analysis of Recombinant UreA-His and Its Mutants

The secondary structures of purified UreA-His and the mutants were detected using a circular dichroism spectrometer (Chirascan, Applied photophysics) to confirm that the changed sites did not affect the general structures of the proteins. The assays were performed as follows: purified recombinant proteins dissolved in 20 mM Tris-HCl (pH 8.0) to a concentration of 0.1 mg/ml were placed in a quartz cell with a path length of 2 mm. CD spectra were measured at wavelengths of 190–260 nm with a bandwidth of 2 nm. Raw CD data were converted into mean residue ellipticity to calculate the secondary structures of the proteins.

### Surface Plasmon Resonance (SPR) Analysis

Affinity studies between Hsp60 and UreA-His were conducted and analyzed using a BIAcore T100 instrument (GE Healthcare). According to the manufacturer’s instruction, interaction affinity is usually determined in two independent ways using Biocore systems: calculation from kinetic constants and measurement of steady-state binding levels. Here, we chose kinetic analysis to calculate the affinity because the association and disassociation of our analyte and ligand are not fast enough to reach the steady state in a short time. And also, this method can provide more information about the interaction, such as the stability of the interaction complex.

Before analysis, all samples were filtered through a 0.2 μm filter followed by centrifugation at 10,000 rpm for 1 min to eliminate trapped air. Running buffer was further degassed by sonication for 20 min each day. Recombinant Hsp60 was immobilized on a BIAcore CM5 sensor chip (GE Healthcare). Binding reactions were performed in phosphate buffered saline (PBS, pH 7.4) at a flow rate of 30 μl per min at 25°C. The analytes (90 μl each of UreA-His or its mutants in PBS buffer) were injected into flow cells 1 and 2 and the association between analyte and ligand was recorded. The adsorbed proteins were removed by injecting 45 μl of 5 mM NaOH. Sensorgrams were recorded and analyzed with Biacore T100 Evaluation Software. The reaction surface data minus the reference surface data and data corresponding to a blank injection with buffer alone were globally fitted to the Lagmuir model for 1:1 binding (Son et al., 2012). As the affinity between Hsp60 and other proteins is not available, there is no positive control in this experiment. However, our system exhibits different affinities of the mutants with Hsp60 and no binding between UreB-His and Hsp60 (data was not shown), which indicated that it is in a good condition.

## Results

### Impact of Hsp60 on Urease Activity

**Figure 1:**
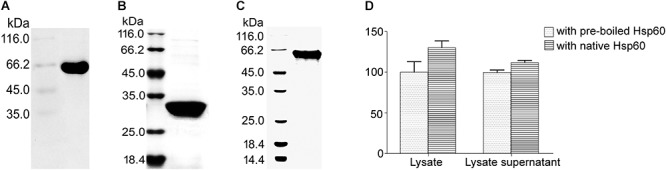
Impact of Hsp60 on the urease activity. **(A)** Purification of recombinant Hsp60 fused with a 6 × His tag on its C-terminus. **(B)** UreA fused with a His tag on its C-terminus. **(C)** UreB fused with a His tag on its C-terminus. The purification of proteins fused with His tags was performed routinely: The supernatant of disrupted bacteria was passed through the Ni-NTA resin at a flow rate of 0.5 ml/min. After being washed with Tris-HCl (50 mM, pH 8.0), the beads were eluted with 250 mM imidazole. For Hsp60, it was further purified with Sephadex G-75 resin at a flow rate of 1 ml/min in HEPES buffer (for urease activity assay) or Tris-HCl (for pull-down assay), or PBS buffer (for SPR analysis). **(D)** Interaction confirmation by examining the impact of Hsp60 on urease activity of *Helicobacter pylori* lysate. *H. pylori* cells were washed and subsequently resuspended in 50 mM HEPES buffer (pH 7.5) to 10^9^ CFU/ml for sonication. The lysate (containing unbroken cells and membrane fractions) or the lysate supernatant (90 μl) was mixed with 100 μl of Hsp60 (0.2 mg/ml), followed by incubation at 37°C for 30 min. Then 100 μl of urea solution (62.5 mM in HEPES buffer) was added and incubated for another 30 min at 37°C. The reaction was stopped by adding 375 μl of regent **A** (containing 10 g/l phenol and 50 mg/l sodium pentacyanonitrosylferrate(III) dihydrate) and 375 μl of regent **B** [containing 5 mg/ml sodium hydroxide, 0.044% (v/v) sodium hypochlorite] successively. After a further 30-min reaction at 37°C, the absorbance at 620 nm was measured. The activity of lysate or lysate supernatant with pre-boiled Hsp60 was taken as control (100%). All experiments were repeated at least three times.

In order to confirm that there is an interaction between Hsp60 and urease, the impact of Hsp60 (with a C-terminal His tag, Figure 1A) on the urease activity was detected by phenol-hypochlorite method. It was found that the recombinant urease (1:1 mixture of UreA-His and UreB-His, Figure 1B,C), either with or without Hsp60, did not exhibit urease activities. This confirmed the conclusion from Maier et al. (2007) that except for structural proteins UreA and UreB, a battery of accessory proteins, such as UreE, F, G, H, are also needed for maturation or activation of urease.

When the recombinant urease was changed to *H. pylori* lysate, obvious urease activities were detected from both Hsp60-treated samples and -untreated samples. And higher activities were obtained from both crude lysate and lysate supernatant samples (Figure 1D). This result reveals an interaction between Hsp60 and urease and supports Hsp60 as the molecular chaperone of urease.

### Expression and Purification of Recombinant Proteins for Pull-Down Assays

**Figure 2:**
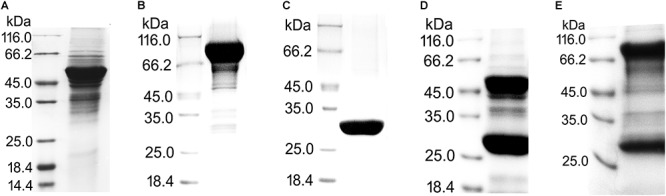
Purification of recombinant proteins with GST tags for pull-down assays. **(A)** UreA fused with GST tag on its N-terminus (GST-UreA). **(B)** UreB fused with GST tag on its N-terminus (GST-UreB). **(C)** GST tag. **(D)** UreA fused with GST tag on its C-terminus (UreA-GST). **(E)** UreB fused with GST tag on its C-terminus (UreB-GST). The recombinant proteins with GST tags were all purified as follows: The bacterial cells were disrupted in Tris-HCl (50 mM, pH 8.0) and centrifuged at 11,000 rpm for 30 min. The supernatant was then incubated with glutathione Sepharose 4B beads overnight with gentle inversion. Next, the beads were washed three times with Tris-HCl (50 mM, pH 8.0) and eluted with glutathione (reduced form) to a final concentration of 10 mM. During the purification of UreA-GST and UreB-GST, a large amount of cleaved GST tag (about 26 kDa) was also eluted from the beads.

**Figure 3:**
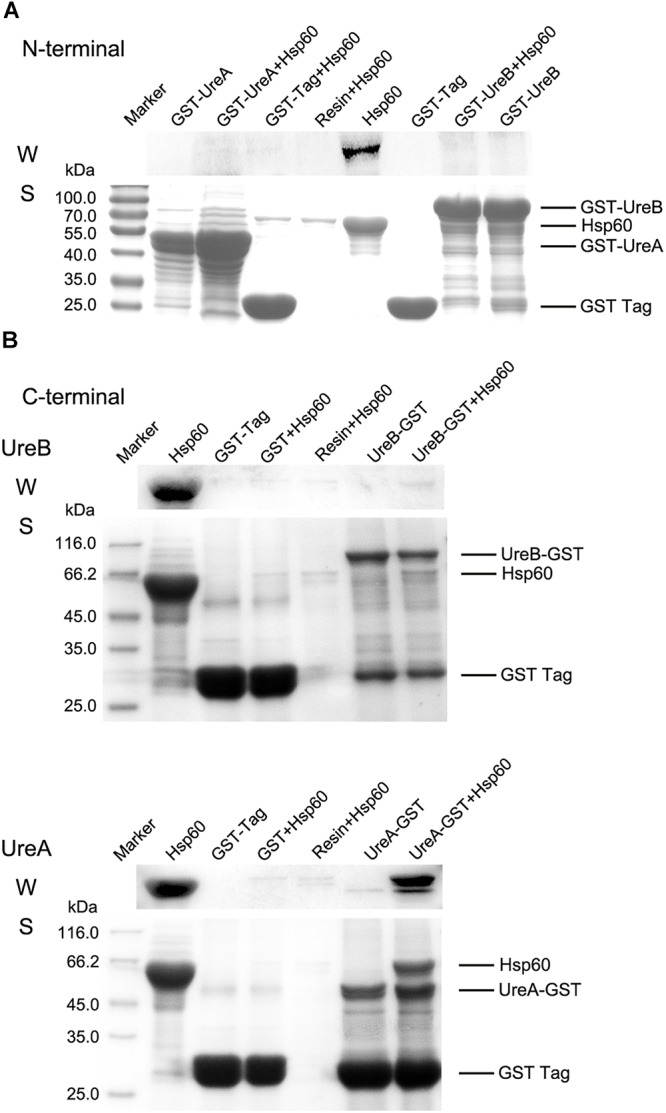
Pull-down assay. **(A)** Results of GST pull-down assay to detect the interaction of GST-UreA and GST-UreB with Hsp60. **(B)** The interaction of Hsp60 with UreA-GST or UreB-GST was verified individually. All pull-down assays were performed as follows: 20 μl of glutathione Sepharose 4B beads saturated with GST-fused proteins were incubated with 0.4 ml of Hsp60 (1 mg/ml dissolved in 50 mM Tris-HCl, pH 8.0) at 4°C for 2 h. The pellets were washed four times and resuspended in SDS-PAGE sample buffer for Western blotting (W) and SDS-PAGE (S) analysis. Hsp60 co-incubated with GST-tag immobilized beads and empty beads were used as negative controls. Purified Hsp60 was used as a positive control. Anti-His antibody was used to detect the bound Hsp60 and horseradish peroxidase (HRP)-conjugated goat anti-mouse IgG 158 was used to detect the primary antibody. All experiments were repeated at least three times.

In total, five proteins fused with different tags were expressed in *E. coli* for pull-down assays (Figure 2). To exclude a stereo-hindrance effect of the GST tag, recombinant UreA and UreB with both N-terminal (GST-UreA, GST-UreB) and C-terminal (UreA-GST and UreB-GST) GST tags were constructed. After purification, GST-UreA, GST-UreB, and the GST tag alone were purified with sufficient qualities (Figure 2A–C). Whereas in the eluates of UreA-GST and UreB-GST, a large quantity of cleaved GST tag was detected (Figure 2D,E). This indicated that UreA-GST and UreB-GST were not as stable as GST-UreA and GST-UreB, and the tag had been cleaved or not incorporated into the final product. As no interaction was detected between the GST tag and Hsp60 in our previous experiments (also shown in Figure 3, GST tag as a negative control), these GST tags were not removed by further efforts.

### Pull-Down Assays of Hsp60 by GST Fused UreA and UreB

To further substantiate the interaction between Hsp60 and urease, a GST pull-down assay was performed. The heterologously expressed N/C terminal GST fused UreA, UreB, or the GST tag alone were immobilized on glutathione beads and subsequently incubated with purified Hsp60. Then, both Western blotting and SDS-PAGE were performed to determine the binding of Hsp60 to the beads (Figure 3). When the GST tag was fused to the N terminus of either UreA or UreB, no obvious band with a size of Hsp60 was pulled down from the incubated solution in either Western blot or SDS-PAGE result (Figure 3A). In Figure 3B, UreB-GST did not interact with Hsp60 either. However, when UreA was fused with GST tag to its C terminus, an obvious band corresponding to the size of Hsp60 was detected in samples eluted from UreA-GST-immobilized beads, but not in other negative control lanes. From the SDS-PAGE results, it could be seen that the amount of UreA-GST is approximately equal to that of Hsp60. Quantitative analysis of these two bands by Image J software showed that the amount of Hsp60 and UreA-GST is 1:1.23, which indicated a 1:1 binding. These results revealed that Hsp60 interacts directly with UreA rather than UreB. The reason that GST-UreA did not pull down Hsp60 while UreA-GST did, is possibly related to the location of the interaction site. As the GST tag is rather large, with a mass of approximately 26 kDa, we speculate that its fusion blocked the interaction sites in GST-UreA. Therefore, it implies that the interaction site is near the N-terminus of UreA.

### Homology Modeling of Hsp60 in *H. pylori*

The homology model of Hsp60 was generated using I-TASSER using GroEL from *E. coli* (PDB: 2YNJ) (Bartesaghi et al., 2012) as a template. After modeling, several scores for estimating the quality of the predicted model were generated. The confidence score (C-score) is one of the most important parameters. Typically, a C-score ranging from -5 to 2 is acceptable, where a higher value signifies a model with higher confidence (Roy et al., 2010). For the model of Hsp60 that we constructed, its C-score was 0.81, revealing it a good model. Based on the C-score, the template modeling score (TM-score) and root mean square deviation (RMSD value) of Hsp60 were predicted to be 0.82 ± 0.08 and 5.9 ± 3.7 Å (A TM-score > 0.5 indicates a model with correct topology while RMSD is the average distance of all residue pairs between the model and the template).

**Figure 4:**
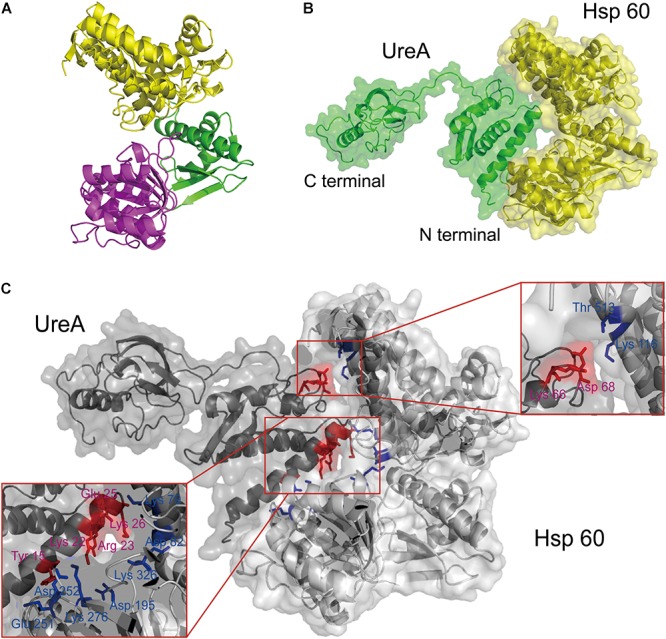
The interaction analysis between UreA and Hsp60 by molecular docking. **(A)** Structure of the constructed Hsp60 monomer from *H. pylori* based on *E. coli* GroEL (PDB: 2YNJ). **(B)** The interaction model of UreA (PDB: 1E9Z) and Hsp60 established by molecular docking. **(C)** Interfaces and key residues analysis. Residues on UreA participating in the interaction are colored in red and noted with purple words. Residues on Hsp60 participating in the interaction are colored and noted in blue.

The result visualized using PyMOL (Figure 4A) indicated that an *H. pylori* Hsp60 monomer is made up of 19 α-helices, 17 β-sheets and several random coils, which form three domains arranged like a reversed “C.” The largest domain (the yellow region, Figure 4A) was made up of more than 240 residues from the N- and C-terminus. This domain is rich in α-helices and well ordered. When assembled into a chaperone cylinder, according to the functional analysis of its template (Braig et al., 1994), it locates at the equator of the polymer serving as the foundation for the chaperonin structure. The central part of the sequence (residues 191–376) forms another domain (the magenta region), which contains equivalent helices, β-sheets and random coils. This region would form the opening of the central cavity (Braig et al., 1994). The intermediate domain is the smallest (the green region, totaling 89 residues), which provides a covalent connection between the other two domains.

### Molecular Docking Simulation and Interface Analysis

Pull-down analyses showed that Hsp60 is a UreA binding protein. Molecular docking was subsequently performed between these two proteins. The weighted binding free energy of the selected cluster center was -1034.3 scored by ClusPro. The model revealed the potential Hsp60-UreA binding orientation, where the N-terminus of UreA is embedded in the “C” cavity of Hsp60 (Figure 4B).This is consistent with the result that the UreA-GST could pull down Hsp60 while GST-UreA could not.

Further analysis suggested that hydrogen bonding and salt bridge are the main forces leading to the association of UreA:Hsp60. Under these forces, two stable interfaces were formed on which seven residues of UreA were predicted to interact with Hsp60 (Figure 4C). The main interface was formed between the first α-helix of UreA and the bottom inner-side of the “C” cavity of Hsp60. On this interface, five residues of UreA, including Y15, K22, R23, E25, and K26, were predicted to interact with E251/D252/K276, D195, D195, K79, and D82/K326 on Hsp60, respectively. The other interface was formed between K66, D68 on UreA and T513, K116 on Hsp60. In total, the size of the interface area is 874.4 Å^2^, calculated by PDBePISA server^2^.

### Expression of Site-Directed Mutants of UreA-His

**Figure 5:**
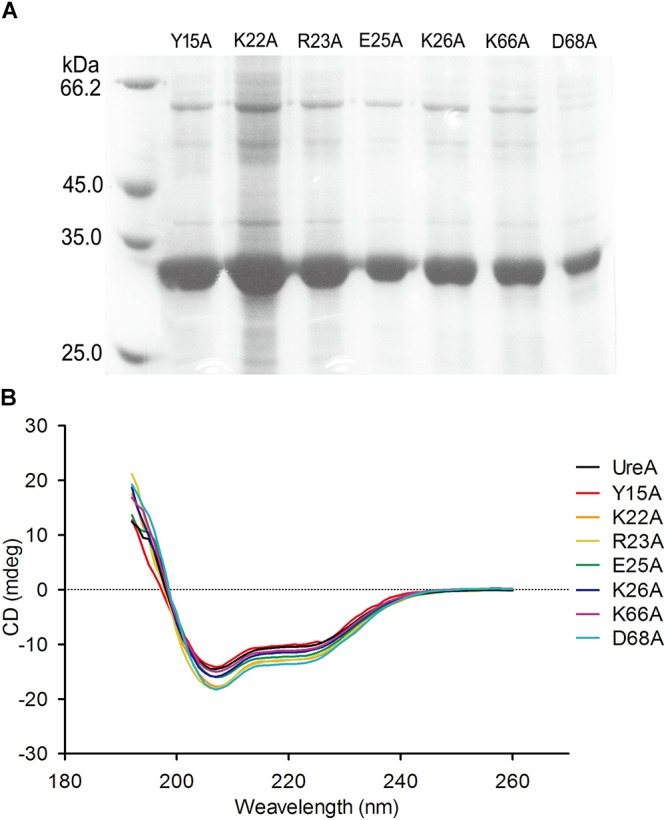
Purification and secondary structural analysis of UreA-His mutants. **(A)** Seven purified mutants of UreA-His. **(B)** CD spectra of UreA-His and its mutants. UreA-His mutants were purified in the same procedure as Hsp60, except that the buffer used was changed to PBS (pH 7.4).

To assess the importance of the predicted residues in the interaction process, we designed the following site-directed mutants based on UreA-His expression system: Y15A, K22A, R23A, E25A, K26A, K66A, and D68A. The purity of these proteins is shown in Figure 5A. Comparison of the CD spectra of UreA-His and the mutants indicated that mutation did not cause significant structural changes in UreA (Figure 5B).

### Surface Plasmon Resonance (SPR) Analysis

**Figure 6:**
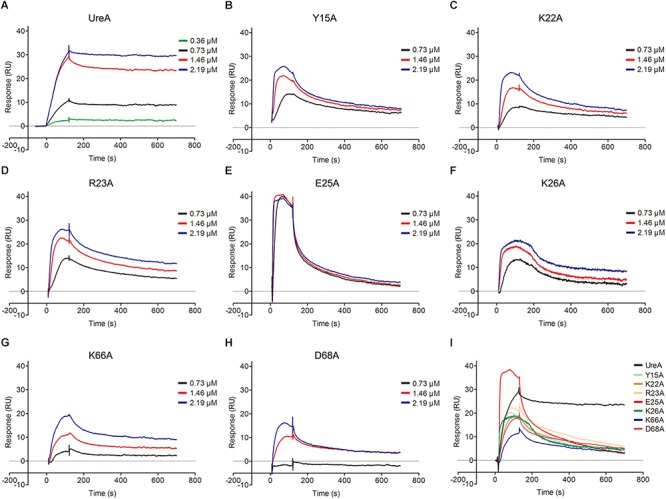
Sensorgram data of UreA-His and its mutants binding to Hsp60. **(A–H)** Wild-type UreA-His or its mutants with a series of concentrations (0.36, 0.73, 1.46, and 2.19 μM) were injected over a control surface (no Hsp60) and a surface containing ∼600 RU of Hsp60 for 120 s with the dissociation monitored for 800 s. Then, the analyte was removed by injecting 45 μl of 5 mM NaOH in 90 s to regenerate the surface. For the mutants, only three protein concentrations (0.73, 1.46, 2.19 μM) were retained in the graph. **(I)** Integrated sensorgram data of UreA-His and its seven mutants binding to Hsp60 with a concentration of 40 μg/ml (1.46 μM). All experiments were repeated at least three times.

Pull-down assays and molecular docking analyses strongly suggested that Hsp60 could bind to the N-terminus of UreA. To further quantitatively explore the interaction of Hsp60 with UreA and determinate the residues that play key roles in the interaction, SPR was performed. To get more accurate results, a series of sensorgrams, obtained at different concentrations of UreA-His binding to the Hsp60 surface, were analyzed to calculate the association (k_on_) and dissociation (k_off_) rate constants (Figure 6). Values of k_on_, k_off_, and KD (k_off_/k_on_) for Hsp60 binding to UreA-His and its mutants are given in Table 2. There is a high affinity between wild type UreA-His and Hsp60 (KD = 17.1 nM). All seven mutants exhibited weaker affinities (higher KD values) than wild-type UreA-His. These results suggested that the predicted interfaces are correct and all predicted residues participate in the interaction. Further analysis of the k_on_ and k_off_ values of the mutants revealed that higher KD values were mostly caused by faster dissociation rates (higher k_off_ values) of the interaction complexes (as the association of most of the mutants did not exhibit obvious changes). Among these mutants, D68A showed the weakest affinity for Hsp60. This affinity attenuation results from both a dramatic decrease in the association rate and an increased dissociation rate. Therefore, the ionic bond between D68 on UreA-His and K116 on Hsp60 appears to play the most important role in binding.

**Table 2:** Values of k_on_, k_off_, and KD (k_off_/k_on_) for Hsp60 binding to UreA-His and its mutants.

Proteins	k_on_ (M^-1^s^-1^)	k_off_ (s^-1^)	KD (M)
UreA-His	7286 ± 15	1.26E-04 ± 1.1 E-06	1.7E-8
Y15A	11240 ± 287	1.64 E-03 ± 2.8E-05	1.5E-7
K22A	5836 ± 146	1.79E-03 ± 2.0E-05	3.1E-7
R23A	5068 ± 85	1.44E-03 ± 2.6E-04	2.8E-7
E25A	40220 ± 1667	5.67E-03 ± 6.5 E-05	1.4E-7
K26A	26200 ± 250	1.22E-02 ± 4.6E-05	4.6E-7
K66A	5397 ± 214	1.15E-03 ± 2.9E-05	2.1E-7
D68A	1156 ± 41	2.27E-03 ± 2.5 E-04	2.0E-6

Further analysis indicated that, in contrast to the other five mutants with limited changes in the k_on_ values, E25A and K26A bind to Hsp60 with a much faster association rate than wild-type UreA-His (40220/26200 to 7286). Meanwhile, the dissociation rates of these two mutants were also greatly increased. Therefore, when we integrated the sensorgrams of different mutants obtained at the same concentration (40 μg/ml, 1.46 μM) together, the curves of E25A and K26A mutants are steeper than the others (Figure 6I). Faster association and dissociation, although with limited changes to the KD values, suggested that E25A and K26A also play important roles in the interaction.

Taken together, the SPR analysis clearly confirmed the interfaces predicted by docking and highlights the importance of residues D68, E25, and K26 on UreA and their interactants K116, K79, and D82/K326 on Hsp60 for UreA-Hsp60 binding.

## Discussion

A lot of evidence pointed to Hsp60 as a molecular chaperone of urease in *H. pylori* (Dunn et al., 1991; Austin et al., 1992; Evans et al., 1992; Suerbaum et al., 1994; Ha et al., 2001). However, their interact pattern remains unclear. In this study, we detected the impact of Hsp60 on the urease activity of *H. pylori* lysate, which supports that Hsp60 acts as a chaperone of urease. Then we identified interaction between Hsp60 and the subunits of urease, α or β, by pull-down assay. This experiment revealed that either GST-UreB or UreB-GST could not pull down Hsp60. For UreA, when the GST tag was fused to its N-terminus, it could not pull down Hsp60. However, when GST was fused to its C-terminus, there was an approximately equal amount of Hsp60 detected on the UreA-immobilized beads. These results indicated that Hsp60 interacts with UreA but not UreB. GST-UreA could not interact with Hsp60, probably because the GST tag provides large steric hindrance and affects the interaction between UreA and Hsp60. This also suggested that the interaction sites of UreA are near the N-terminus of the sequence.

To validate the interaction site between UreA and Hsp60, an interaction model was predicted by molecular docking. It was found in this model that the N-terminus of UreA inserted into the cavity of Hsp60 perfectly and generated two interfaces. From the major interface, five residues of UreA were speculated to be within a distance that allows the formation of interaction forces with Hsp60. While on the other interface, another two interacting residues were identified. These potentially interacting residues on UreA were substituted to alanine individually by site-directed mutagenesis to verify their roles in the interaction. SPR analysis revealed that the association rate of wild-type UreA-His and Hsp60 was relatively fast and that the dissociation rate was very slow, meaning that there is a strong affinity between these proteins. After mutagenesis, all mutants exhibited weaker affinities for Hsp60 than the wild-type UreA-His. CD spectra indicated that mutation did not cause structural changes. Therefore, the weak affinities of the mutants mainly resulted from the mutation of amino acids rather than structural changes. This confirmed that the predicted interfaces were credible. Among these residues, the mutation of D68 caused the greatest attenuation of interaction affinity by dramatically decreasing the association combined with an increase of the dissociation rate. This suggested that D68 plays an important role in the interaction process. Further analysis showed that substitution of E25 or K26 by alanine could dramatically speed up both the association and dissociation rate. Considering that these two residues are located at the forefront when UreA enters the cavity of Hsp60 (Figure 4C), it is speculated that mutation of these residues to alanine would reduce the resistance of the association and speed up binding. Meanwhile, loss of the binding force formed between them and their interacting residues on Hsp60 (K79 and D82/K326) would make the interaction complex unstable. This result implied that K79 and D82/K326 on Hsp60 are important residues that stabilize the binding of UreA.

This study clarifies the interaction between urease, an important pathogenic factor of *H. pylori*, and its molecular chaperone Hsp60. The residues playing key roles in the interaction between UreA and Hsp60 were further studied here. These results lay a foundation for revealing the assembly and maturation mechanism of *H. pylori* urease.

## Ethics Statement

This article does not contain any studies involving human participants or animals.

## Author Contributions

XJ, HZ, and BL conceived and designed the experiments. HZ, YW, XJ, ZX, RM, YD, XB, QR, and YZ performed the experiments. HZ, XJ wrote the manuscript. HZ, XJ, and BL revised the manuscript. All authors approved the final manuscript.

## Conflict of Interest Statement

The authors declare that the research was conducted in the absence of any commercial or financial relationships that could be construed as a potential conflict of interest.

## Footnotes

1 http://zhanglab.ccmb.med.umich.edu/I-TASSER/

2 http://www.ebi.ac.uk/msd-srv/prot_int/pistart.html
